# Novel balloon compression-assisted endoscopic injection sclerotherapy and endoscopic variceal ligation in the treatment of esophageal varices: a prospective randomized study

**DOI:** 10.1007/s00464-022-09412-6

**Published:** 2022-07-25

**Authors:** Qianqian Zhang, Jing Jin, Fumin Zhang, Yi Xiang, Wenyue Wu, ZeXue Wang, Derun Kong

**Affiliations:** 1 Department of Gastroenterology, Key Laboratory of Digestive Diseases of Anhui Province, The First Affiliated Hospital of Anhui Medical University, 218 Jixi Road, Hefei, 230022 Anhui China; 2 Fuyang Hospital of Anhui Medical University, Fuyang, Anhui 236000 People’s Republic of China

**Keywords:** Balloon compression-assisted endoscopic injection sclerotherapy, Endoscopic injection sclerotherapy, Endoscopic variceal ligation, Esophageal variceal bleeding, Esophageal varices, Cirrhosis

## Abstract

**Background:**

Herein, our group designed a novel technology, termed balloon compression-assisted endoscopic injection sclerotherapy (bc-EIS), which was applied to improve the efficiency of eradicating esophageal varices (EVs). The present study aimed to compare the rate of eradication and efficacy between bc-EIS and endoscopic variceal ligation (EVL) in the management of EVs.

**Methods:**

Ninety-five patients with esophageal variceal bleeding (EVB) were randomly assigned to receive bc-EIS or ligation alone. Additional treatment sessions were held 1 month later and then at 3-month intervals until eradication of the varices was achieved. Endoscopic follow-up examinations were carried out at 6-month intervals in the absence of recurrence or immediately if there was any recurrent bleeding.

**Results:**

The mean physical injection points per session were 2.89 ± 0.79, and the mean volume of lauromacrogol used per session was 17.74 ± 7.09 ml in the bc-EIS group. The mean band per session was 6.13 ± 0.86. The rate of eradication after one to three rounds of bc-EIS was obviously higher than that of the EVL group (89.36%, 97.87%, and 100% vs. 37.5%, 43.75%, and 47.92%, respectively). Retrosternal pain or discomfort in the bc-EIS group was slightly lower than that in the EVL group (23.4%, 11/47 vs. 31.25%, 15/48). Two and five patients showed mild abdominal bloating and distension between the bc-EIS and EVL groups, respectively (2/47, 4.26% vs. 5/48, 10.42% *P* > 0.05). Nausea and vomiting were reported in one patient (1/47, 2.13%) in the bc-EIS group and three patients (3/48, 6.25%) in the EVL group. However, there were no statistically significant differences between the two groups (*P* > 0.05). No fatal or severe complications, such as esophageal perforation, esophageal stricture or ectopic embolism, were observed.

**Conclusion:**

The bc-EIS method was effective in eradicating EVs and was accompanied by fewer complications.

**Supplementary Information:**

The online version contains supplementary material available at 10.1007/s00464-022-09412-6.

Esophageal varices (EVs) can be caused by portal hypertension. It is a potentially life-threatening clinical condition that demands rapid and efficient treatment. Both endoscopic variceal ligation (EVL) and endoscopic injection sclerotherapy (EIS) can be used as efficient endoscopic treatments for EVs. The first-line endoscope treatment, as with EVL, is a standard pattern for patients complicated by EVs.

According to a study performed by Krige et al*.* [1], EVL seems not to be superior to EIS in terms of the lower rate of variceal eradication. EVL eradicates superficial varices through mechanical strangulation with rubber bands [2] but cannot achieve complete eradication of the interconnecting perforating and feeder vessels in the deeper esophageal wall layers [3]. Additionally, mucosal fibrosis and scarring induced by repeated EVL procedures limit the pliability of the mucosa and preclude further successful banding application [1]. In contrast, EIS can be conducted either intravariceally or paravariceally to obliterate varices by thrombosing the veins or by thickening the mucosa overlying the veins in this area, respectively [4]. However, EIS is not without drawbacks. The intravariceally injected sclerosant may flow out to the drainage vein, thus impairing the effectiveness of EIS and increasing the risk of ectopic embolism through venae intercostales and venae azygos. Based on the disadvantages of EIS, a novel technology, termed balloon compression-assisted endoscopic injection sclerotherapy (bc-EIS), was developed. A previous case reported a novel technology to improve the therapeutic efficacy of the sclerosing agent and to increase the residence time of the sclerosing agent [5]. The aim of the present study was to assess the rate of eradication and efficacy of bc-EIS in comparison with EVL for EVs in a prospective cohort.

## Materials and methods

### Patients and general management

This cohort study was performed on patients managed in the First Affiliated Hospital of Anhui Medical University from April 2019 to April 2021.

Patients presenting with an episode of upper gastrointestinal bleeding (UGIB, haematemesis, or melena or both) were admitted to the hospital and underwent endoscopy as soon as they had been admitted. Patients were treated by EVL or bc-EIS if they were actively bleeding at the time of endoscopy or had red colour signs of recent haemorrhage. Somatostatin (250 µg/h; Hybio Co., Ltd.) or octreotide (50 µg/h; Suzhou Tianma Specialty Chemicals Co., Ltd.) was started before endoscopy and continued for 3 days after treatment and repeated if there was further bleeding, as well as intravenous antibiotics and prophylaxis for hepatic encephalopathy were managed. The inclusion criteria were as follows: (i) 18 ≤ age ≤ 80 years; (ii) liver cirrhosis was diagnosed according to imaging and pathology examination, EVs were diagnosed according to clinical manifestations, and endoscopy, primary prevention, or secondary prevention were proposed; and (iii) the patient signed a preoperative informed consent form.

The exclusion criteria were as follows: (i) age < 18 or > 80 years; (ii) bleeding from fundal varices of the stomach or sources other than esophageal varices; (iii) poor overall health status, including advanced hepatocellular carcinoma, hepatic encephalopathy grade III and IV, and malignancies other than hepatocellular carcinoma meeting the Milan criteria [6]; (iv) massive bleeding resulting in death before randomization; and (v) refusal to participate in the study and different medical therapies compared to the previous method (EVL or bc-EIS).

At the time of endoscopy, patients meeting the inclusion criteria were randomized to undergo bc-EIS or EVL based on computerized random digit allocation. Cirrhosis was diagnosed or suggested by liver biopsy or imaging study and clinical assessment.

### Follow-up

EVL or bc-EIS treatment was repeated every month as long as the patients were in good condition until the complete eradication of EVs. Follow-up endoscopy was performed 3 months after the eradication of EVs and then every 6 months in the absence of recurrence. They were also examined regularly every month and in between as needed. In case of rebleeding, the patient was admitted to the hospital and underwent endoscopy as soon as possible to determine the source of bleeding. EVs were considered the source of bleeding if active bleeding or signs of recent haemorrhage were seen on endoscopy or if only the varices were seen in the absence of other visible mucosal lesions. Everyone was followed up in case of bleeding until the last follow-up or death. The same bc-EIS or EVL procedures were repeated in the case of EV recurrence.

The study was approved by the hospital ethics committee and all the patients consented. All procedures followed were performed according to the ethical standards of the responsible committee on human experimentation (institutional and national) and with the Helsinki Declaration. This study was registered at Clinical Trial.gov (no. ChiCTR2000039974).

### Definitions of variceal eradication, recurrence, and rebleeding

Total variceal eradication was defined as the disappearance of varices after treatment, including thrombosed varices [7]. Variceal recurrence was defined as the reappearance of eradicated varices on endoscopy [1]. The final assessment of variceal eradication or recurrence had to be agreed upon by two experienced endoscopists. Variceal rebleeding was defined according to the Baveno IV consensus [8].

### Technique of balloon compression-assisted endoscopic injection sclerotherapy and endoscopic variceal ligation

#### Balloon compression-assisted endoscopic injection sclerotherapy

**Fig. 1:**
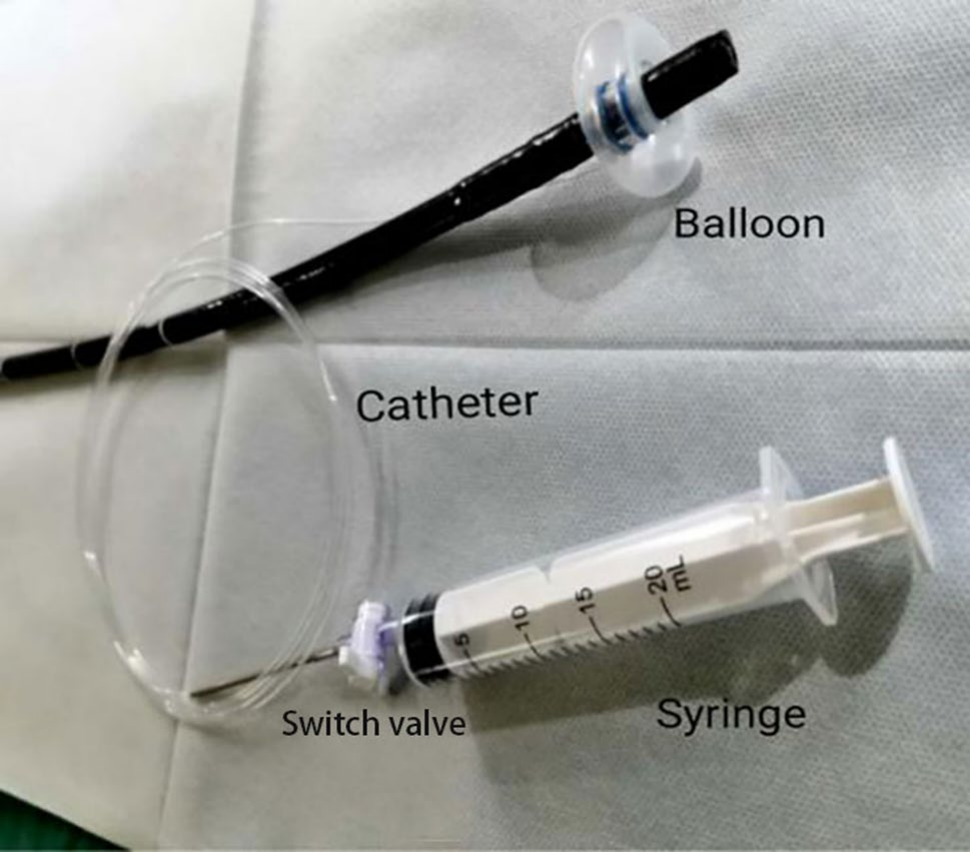
The initial outer diameter of the balloon was 1.3 cm, which could expand to a maximum of 4 cm after inflation with 30 ml air

**Fig. 2:**
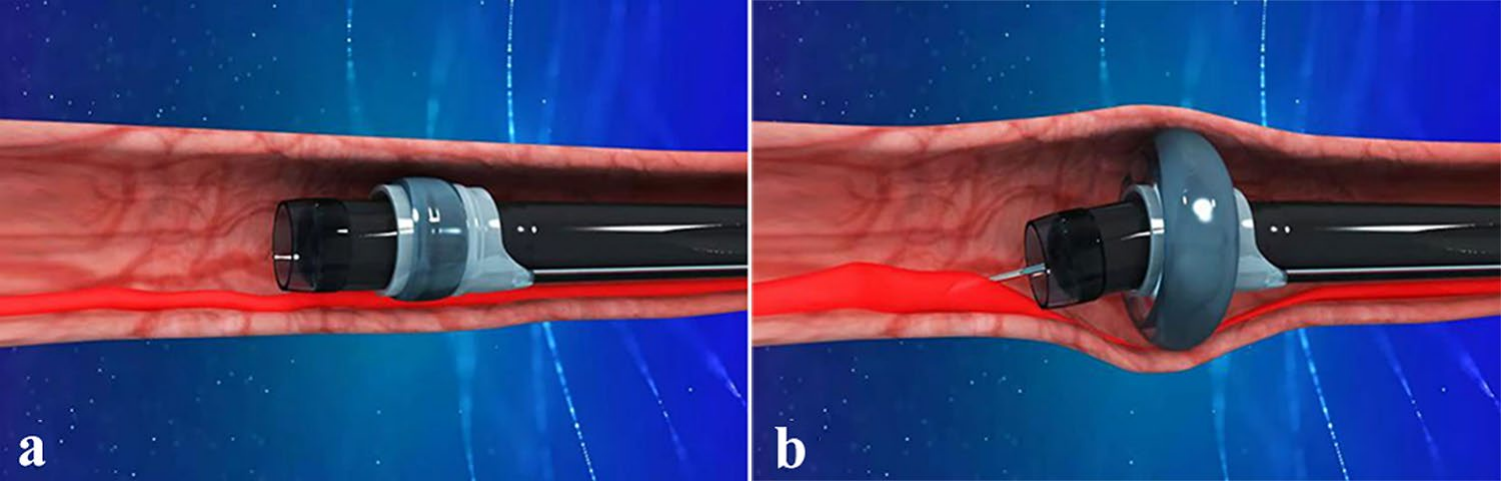
**a** The lubricated endoscope together with the deflated balloon and the transparent cap were inserted into the lumen of the oesophagus. **b** The varicose veins could be fully compressed by the inflated balloon, a disposable endoscopic injection needle was introduced through the operation channel and injected into the base of the variceal columns near the cardia

**Fig. 3:**
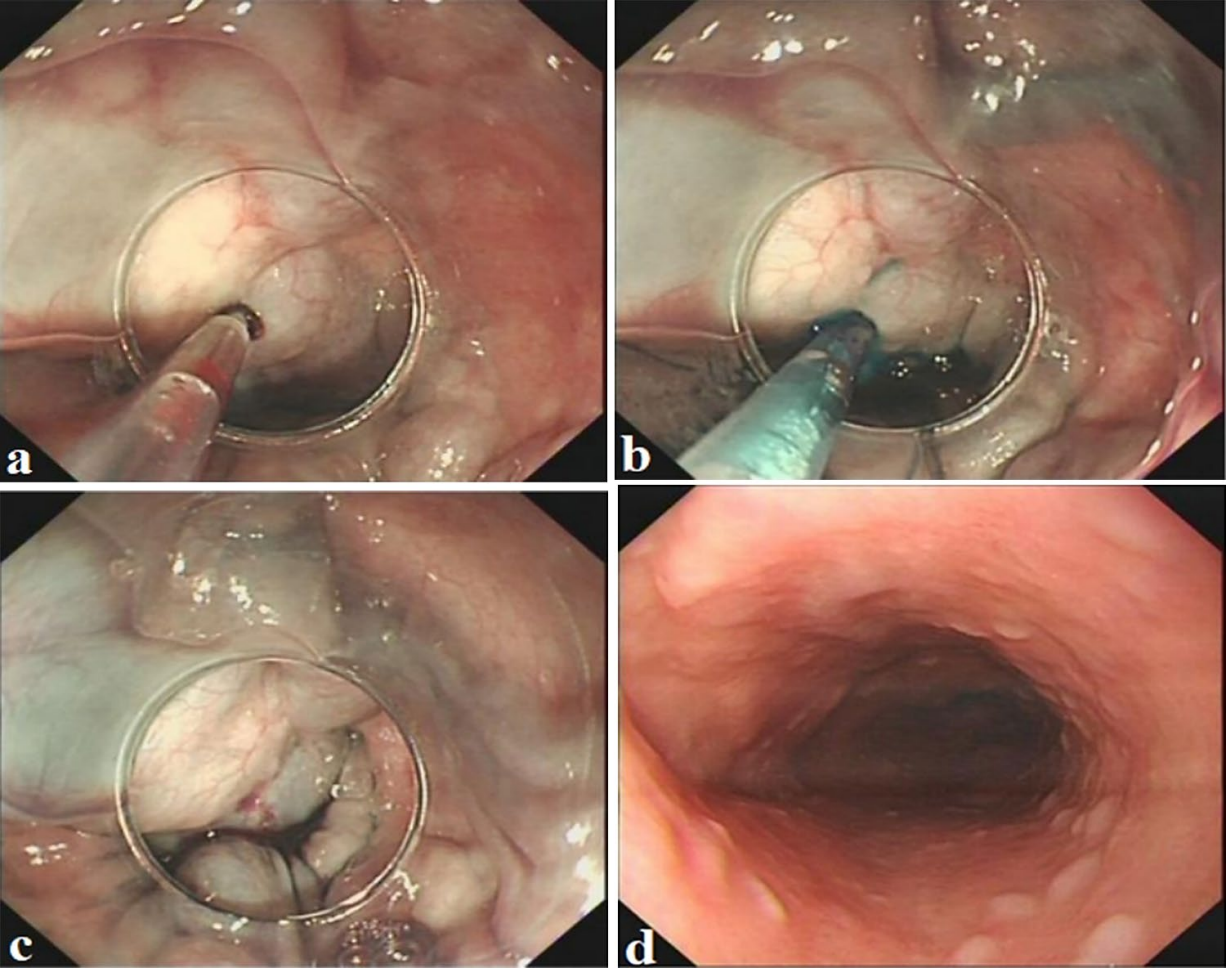
**a** Blood flowed back into the needle. **b** Lauromacrogol was administered intravaliceally, which was mixed with methylthioninium chloride as tracers. **c** The injection point was compressed by a needle sheath or transparent cap. **d** The esophageal varicose vein disappeared after treatment

Full details of the balloon compression-assisted endoscopic injection sclerotherapy used have been published previously [5]. An inflatable balloon (Jiangsu Vedkang Corporation, VKD-QN-16-160/180-A) for bc-EIS was prechecked to ensure airtightness, which was fixed over an endoscope (GIF Q260J; Olympus Corporation) at a distance of 3 cm away from its distal end. The length and inner diameter of the balloon were 1.6 and 1.1 cm, respectively. The initial outer diameter of the balloon was 1.3 cm, which could expand to a maximum of 4 cm after inflation with 30 ml air (Fig. 1). Subsequently, a transparent cap (MAJ-290; Olympus Corporation) was placed at the distal end of the endoscope. The lubricated endoscope together with the deflated balloon and the transparent cap were inserted into the lumen of the oesophagus (Fig. 2a). A total of 20 ml air were next injected into the balloon through a thin catheter, causing its outer diameter to expand to 3.5 cm when the end of the endoscope was close to the target varices. Subsequently, a disposable endoscopic injection needle (NM-400L-0425; Olympus Corporation) was introduced through the operation channel and injected into the base of the variceal columns near the cardia (Fig. 2b). When blood flowed back into the needle, 1% lauromacrogol (10 mg/mL; Tianyu Co., Ltd, Shanxi, China) was administered intravaliceally, which was mixed with methylthioninium chloride (Jumpcan Co., Ltd, Taixing, China) as tracers at a ratio of 10:0.1 (Fig. 3a, b). The needle was rapidly withdrawn once the injection was completed, followed by compression onto the injection point by a needle sheath or transparent cap depending on the severity of immediate bleeding after the injection (Fig. 3c, supplementary material, Video 1). The EVs were compressed by the inflated balloon for 20 min, and the curative effect was evaluated according to lauromacrogol, which is a mixed tracer of methylthioninium chloride. The number of injection sites and the dose of lauromacrogol were determined according to the severity of the varices in an attempt to eradicate the visible varices in one session. The volume of each injection should not exceed 10 ml, and the total volume per session should not exceed 35 ml.

### Endoscopic variceal ligation

Endoscopic ligation was performed with endoscopic ligation devices, and concrete details of the variceal ligation technique were actualized according to previous guidelines [9]. The endoscopic over tube consisted of a plastic sheath 20 mm in diameter and 25 cm long that was inserted into the proximal oesophagus before ligation was performed. The over tube remained in the oesophagus throughout the procedure and facilitated the repeated removal and reinsertion of the endoscope, which was necessary for multiple ligation to be affected. Briefly, varices were ligated individually with multiband ligators (Wilson-Cook Medical Inc., Winston-Salem, NC, USA), starting at or just below the gastroesophageal junction and continuing cephalad to 7 cm above that junction. All individual varices were ligated at least once per treatment, and larger varices were often ligated twice at separate points, one caudad, and one cephalad.

### Statistical analysis

Continuous variables, which were reported as the mean ± SD, were compared using the unpaired Student’s *t* test or the nonparametric Mann–Whitney rank-sum test as the median (interquartile range [IQR]). Categorical variables were compared with Fisher’s exact test. All P values were two tailed, and values of less than 0.05 were considered to indicate statistical significance. Calculations were performed with SPSS statistical software (SPSS 21.0; IBM Corp., Armonk, New York, USA).

## Results

### Patient characteristics in the bc-EIS and EVL groups

Ninety-five patients participated in this study, forty-eight patients were randomly allocated to receive EVL, and forty-seven to receive bc-EIS. All patients were confirmed to have EVs by endoscopy.

There was a male predominance in gender (70 males, 73.68%). Chronic hepatitis B remained the main cause of cirrhosis in the current study. Throughout the follow-up period, the patients were enrolled to be treated of primary aetiology (hepatitis B virus, alcoholic cirrhosis, autoimmune hepatitis, schistosomiasis cirrhosis, and combined) and were continued in addition to the other supportive treatments. The number of patients according to the Child–Pugh classification was as follows: Child–Pugh class A (*n* = 58, 61.05%), Child–Pugh class B (*n* = 35, 36.84%), and Child–Pugh class C (*n* = 2, 2.11%). Their demographic characteristics, clinical features, and outcomes according to endoscopy and pattern of treatment are shown in Table 1.

**Table 1:** Characteristics of patients treated by balloon compression-assisted endoscopic injection sclerotherapy or endoscopic variceal ligation

Characteristic	bc-EIS (*n* = 47)	EVL (*n* = 48)	*P* value
Mean age, year, median (IQR)	53(48,65)	52(48,57)	NS
Sex (M/F), n	28/19	42/6	**0.002**
Weight, kg, (mean ± SD)	63.92 ± 9.35	62.12 ± 9.48	NS
Aetiology, *n*			**0.006**
HBV-DNA related	27	39	
Alcoholic	6	1	
Autoimmunity	3	5	
Schistosomiasis	2	1	
Combined	0	1	
Others	9	1	
Child–Pugh class (A/B/C), *n*	30/17/0	28/18/2	NS
Diabetes mellitus, *n*	7	5	NS
High blood pressure, *n*	7	5	NS
NSSB medication, *n*	2	0	NS
Splenectomy, *n*	8	6	NS
Portal vein thrombosis, *n*	15	12	NS
History of esophageal varicose veins and bleeding, *n*	45	38	**0.015**
Aim, *n*			NS
Control emergency bleeding	14	14	
Primary prevention	2	7	
Secondary prevention	31	27	
Haemoglobin, g/L, median (IQR)	75 (64.90)	91 (64.115)	**0.009**
APTT (sec)	28.7 (26.55,31.85)	32.65 (29.3,39.08)	**0.001**
Creatinine, mmol/L,(mean ± SD)	61.80 ± 16.94	58.73 ± 15.45	NS
Ascites, *n*			** < 0.001**
None	23	40	
Mild	23	7	
Severe	1	1	
Variceal form (F1, F2, F3), *n*	0/34/13	18/30/0	** < 0.001**
Red colour signs, *n*	46	43	NS
Diameter of varices, mm, median (IQR)	12 (10,15)	8 (6,10)	** < 0.001**
Encephalopathy, *n*	0	0	NS
Occurrence of hepatomat, *n*	4	2	NS

*bc-EIS* balloon compression-assisted endoscopic injection sclerotherapy

*EVL* endoscopic variceal ligation

*NSBB medication* Nonselective β-blocker medication

*NS* not significant

Bold values represented *P* < 0.05

### Variceal eradication of the bc-EIS and EVL groups

In the bc-EIS group, eradication of EVs was achieved for the first time in 42 patients, compared to 18 patients in the EVL group (89.36%, 42/47 vs. 37.5%, 18/48; *P* < 0.001). In addition, after the second treatment, the rate of eradication was obviously higher in the bc-EIS group than in the EVL group (97.87%, 46/47 vs. 43.75%, 21/48; *P* < 0.001). Furthermore, bc-EIS showed complete eradication compared with the EVL group (100%, 47/47 vs. 47.92%, 23/48) (As showed in Table 2).

**Table 2:** Outcome of variceal eradication and rebleeding of patients treated by balloon compression-assisted endoscopic injection sclerotherapy or endoscopic variceal ligation

Characteristics	bc-EIS (*n* = 47)	EVL (*n* = 48)	*P* value
Eradication patients, *n* (%)	47 (100%)	43 (89.58%)	NS
Total sclerosant volume, ml (mean ± SD)	17.74 ± 7.09	–	NS
Injection points per session (mean ± SD)	2.89 ± 0.79	–	NS
Rubber bands (mean ± SD)	–	6.125 ± 0.86	NS
Variceal eradication, *n* (%)			** < 0.001**
The first time	42 (89.36%)	18 (37.5%)	** < 0.001**
The second time	46 (97.87%)	21 (43.75%)	** < 0.001**
The third time	47 (100%)	23 (47.92%)	** < 0.001**
Rebleeding, *n* (%)	0 (0%)	2 (4.17%)	NS
Variceal recurrence, *n* (%)	0 (0%)	1 (2.1%)	NS
No. of deaths due to EVB, *n* (%)	0 (0%)	0 (0%)	NS

*bc-EIS* balloon compression-assisted endoscopic injection sclerotherapy

*EVL* endoscopic variceal ligation

*EVB* esophageal variceal bleeding

*NS* not significant

Bold values represented *P* < 0.05

### Recurrent bleeding after variceal eradication

One of the forty-eight patients with recurrent esophageal variceal bleeding (EVB) presented with melena after variceal eradication 2 weeks later. Another patient experienced recurrent bleeding during the regular interval examination at the sixth month after eradication and the same EVL procedure was repeated (As showed in Table 2).

### Complications of the bc-EIS and EVL groups

Possible complications of endoscopic treatment were observed in both groups. The rate of complications in the EVL group was obviously higher than that in the bc-EIS group (47.92%, 23/48 vs. 29.79%, 14/47). Retrosternal pain or discomfort in the bc-EIS group was slightly lower than that in the EVL group (23.40%, 11/47 vs. 31.25%, 15/48). Two and five patients showed mild abdominal bloating and distension between the bc-EIS and EVL groups, respectively (4.26%, 2/47 vs. 10.42%, 5/48 *P* > 0.05). Nausea or vomiting was reported in one patient (1/47, 2.13%) in the bc-EIS group and three patients (3/48, 6.25%) in the EVL group. In addition, there were no statistically significant differences in complications, fever, or embolism. During the regular follow-up, no fatal or severe complications, such as systematic embolic symptoms, including lung, cerebrum, and spleen, were presented. In addition, esophageal stricture, esophageal ulcer, and esophageal perforation were not revealed on endoscopy (As showed in Table 3).

**Table 3:** Complications of patients treated by balloon compression endoscopic injection sclerotherapy or endoscopic variceal ligation

Complications, *n*	bc-EIS (*n* = 47)	EVL (*n* = 48)	*P* value
Retrosternal pain or discomfort	11	15	NS
Abdominal bloating and distension	2	5	NS
Nausea or vomiting	1	3	NS
Esophageal stricture and ulcer	0	0	NS
Esophageal perforation	0	0	NS
Fever	0	0	NS
Embolism	0	0	NS

*bc-EIS* balloon compression-assisted endoscopic injection sclerotherapy

*EVL* endoscopic variceal ligation

## Discussion

Esophageal variceal bleeding (EVB) is a fatal cause of mortality in cirrhotic patients. Since EVB is the principal condition of upper gastrointestinal bleeding and is associated with a high mortality rate, prevention of bleeding might be expected to result in improved survival. Both EVL and EIS are recommended for EVB because of their high safety and efficiency [10–13].

EVL was recommended as the first-line therapy for the prevention and treatment of acute EVB by the gastroenterology and hepatology society guidelines [7]. Approximately, 90% of patients repeatedly treated by EVL achieved the basic functional disappearance of varicose veins, but 20 ~ 75% relapsed within 1 year [14, 15]. In addition, varices of the stomach fundus may be aggravated by the EVL procedure in the condition of the interconnecting oesophagus and gastric vessels [16]. In addition, mucosal fibrosis and scarring emerged by repeated EVL procedures limit the pliability of the mucosa and preclude further successful banding application. It is increasingly difficult to ligate effectively for small varices because of their decreasing size, and EVL is at the risk of band abscission in terms of large varices [17–19].

In traditional EIS treatment methods, sclerosing agents can be introduced into the systemic circulation through venae azygos and intercostal veins; thus, the concentration of sclerosing agents is rapidly reduced. According to previous related studies [16, 20, 21], the amount of sclerosing agent is increased to improve the efficiency in a concentration-dependent manner; however, the possible risk of ectopic embolism is thus increased. It seems to be challenging to improve the concentration and residence time of sclerosing agents in traditional EIS technology.

To improve the efficacy of EIS and to reduce the risk of ectopic embolization, our group developed a novel technology, termed balloon compression-assisted endoscopic injection sclerotherapy (bc-EIS) [5]. It was supposed that with the compression of the proximal oesophagus by an inflated balloon and the effectively accurate intravariceal injection, the sclerosant would tend to be retained at the injection site instead of extending upward and beyond the injection site, thus improving the efficacy of EIS as well as decreasing the risk of ectopic embolism. Based on the novel technology of bc-EIS, the current study performed a randomized, controlled trial to assess the rate of eradication and efficacy of bc-EIS in comparison with EVL as endoscopic therapy for EVs. To our knowledge, this is the first report to compare the two procedures in the treatment of EVs.

Before EIS was conducted according to routine procedures [22], an inflatable balloon for bc-EIS was prechecked to ensure air tightness and fixed over an endoscope at a distance of 3 cm away from its distal end according to the instructions. Subsequently, the lubricated endoscope together with the deflated balloon and the transparent cap were inserted into the lumen of the oesophagus, and a total of 20 ml air were next injected into the balloon through a thin catheter, causing its outer diameter to expand to 3.5 cm when the end of the endoscope was close to the target varices. The initial treatment outcome of the bc-EIS revealed that 1% lauromacrogol (10 mg/mL; Tianyu Co., Ltd, Shanxi, China) was mixed with methylthioninium chloride (at a ratio of 10:0.1), which served as a tracer and was intravariceally administered. Lauromacrogol (1%), which is efficient on lipid molecules in endothelial cells, induces intimal inflammation and thrombosis formation, followed by the formation of fibrotic tissue and obliteration of the targeted vein [23]. In accordance with the instructions of lauromacrogol related to thrombosis formation, the EVs were compressed by the inflated balloon for 20 min to ensure the full action time of the sclerosant and to achieve the optimal curative effect between the sclerosant and the endothelium. The effect of embolism was evaluated with the unfaded sclerosant (mixed with a tracer of methylthioninium chloride), which was a sign of efficient obstruction. Otherwise, a further step was needed to expand the balloon to 30 ml, the balloon diameter was 4.0 cm, and the same procedure was repeated. Furthermore, the efficacious embolization was further certified by the unfaded sclerosant (mixed with the tracer of methylthioninium chloride) following deflated balloon withdrawal from the oesophagus. All of the procedures are not difficult for endoscopists to install the balloon according to the instructions and to evaluate the effect of embolization, even without professional training. In the present study, the mean injection point per session was 2.89 ± 0.79 and the mean volume of lauromacrogol used per session was 17.74 ± 7.09 ml in the bc-EIS group.

Previous studies indicated that the rate of variceal eradication could be successfully realized beyond seventy-nine percent by EVL [3, 24–27]. In terms of the theories of ligation, EVL is plagued by a lower rate of eradication because EVL obliterates varices via mechanical strictures induced by rubber bands, and it is not efficient to obliterate the deeper varices and perforating veins during the first EVL session; EVL is usually associated with 2 or 3 sessions [28]. In contrast, the novel technology of bc-EIS is superior to EVL to eradicate EVs efficaciously owing to the accurate intravariceal injection as well as obliterating the deeper varices and perforating veins, which is not at the risk of band abscission. In the current study, eradication of the varices was achieved at first by bc-EIS in 42 of 47 patients and by EVL in 18 of 48 patients. The rate of variceal eradication in the bc-EIS group at first was obviously higher than that in the EVL group (89.36%, 42/47 vs. 37.5%, 18/48 *P* < 0.001). At the second-time point, 46 patients in the bc-EIS group and 21 patients in the EVL group achieved variceal eradication. The cumulative eradication rate after the second-time treatment by bc-EIS was more effective than EVL (97.87%, 46/47 vs. 43.75%, 21/48 *P* < 0.001). Furthermore, 47 patients in the bc-EIS group and 23 patients in the EVL group achieved variceal eradication. The cumulative eradication rate after the third eradication by bc-EIS was obviously higher than that by EVL (100%, 47/47 vs. 47.92%, 23/48 *P* < 0.001).

In the present study, there were slight differences regarding postoperative complications between the two groups. Mild-to-moderate retrosternal pain or discomfort was reported in approximately 31.25% (15/48) of patients accepting EVL, which is different from that reported in patients who underwent common upper endoscopy procedures (9–18%) [29]. Two and five patients showed mild abdominal bloating and distension between the bc-EIS and EVL groups, respectively (4.26%, 2/47 vs. 10.42%, 5/48; *P* > 0.05). Nausea or vomiting was reported in one patient (1/47, 2.13%) in the bc-EIS group and three patients (3/48, 6.25%) in the EVL group. A previous report suggested that esophageal strictures may be more frequently associated with paravariceal injections, which induce esophageal ulcers [30]. However, in the current study, lauromacrogol was intraventricularly administered and effectively remained in the variceal vessels with the help of balloon compression. Efficient inflammation and thrombosis were directly induced in the venous endothelium owing to intravariceal injections and thus ulcers involving the subesophageal mucosa or the muscle layer were not present. Therefore, the disappearance of EVs was revealed on gastroenterology, and no serious adverse events in the form of esophageal stricture were observed during the follow-up.

Previous cases reported multiple embolisms, including lung, cerebrum, and spleen embolisms, after the injection of N-butyl-2-cyanoacrylate for the treatment of gastroesophageal varices [31, 32]. Mild pulmonary embolism complicates with cough; however, severe or fatal pulmonary embolism, such as dyspnoea and transient hypoxemia, requires auxiliary respiratory support. Cerebral embolism can be complicated with limb hemiplegia, slurred speech, and even coma. Despite the limited reports on ectopic embolism related to EIS during the treatment of EVs, its occurrence can be disastrous. In the present study, the inflated balloon immobilized and compressed the targeted varices, thus decreasing the outflow of sclerosant through collateral veins and reducing the probability of ectopic embolism. However, the diagnosis related to imaging could not be achieved directly owing to the absence of contrast agents as tracers. In our institution, computed tomography angiography (CTA) scanning was regarded as the usual detection method to evaluate the status of spontaneous shunts in every procedure and for better treatment to avoid complications, such as ectopic embolism. In addition, clinical symptoms of ectopic embolism were collected intraoperatively and postoperatively. In the current study, no fatal or severe systematic embolic symptoms, such as cough, haemoptysis, thoracodynia, or hemiplegia of the body, were presented.

The present study was not without drawbacks. This was a prospective single-centre study that included a limited number of cases and was conducted on the basis of a 6-month follow-up. In the future, a large number of cases with long-term follow-up is necessary to discover the differences in the rate of variceal eradication and complications between EVL and bc-EIS.

In conclusion, the intravariceally injected sclerosant with the help of balloon compression assistance can remain in the variceal vessels longer instead of flowing out to the drainage vein, venae intercostales, and/or venae azygos. Thus, the effective concentration was increased comparatively, and variceal vessels were eradicated efficaciously owing to the accurate intravariceal injection. The novel technology of bc-EIS revealed a higher and effective rate than EVL in eradicating EVs.

## Supplementary Information

Below is the link to the electronic supplementary material.

Supplementary file1 (MP4 76346 kb) The procedure of the balloon compression-assisted endoscopic injection sclerotherapy.
